# Books and Magazines

**Published:** 1898-10

**Authors:** 


### Books and Magazines.

ATLAS AND ESSENTIALS OF BACTERIOLOGY.—By Prof. K. B. Lehmann, Chief of the Hygienic Institute in Wurzburg, and Dr. Rudoff Neumann, Assistant in the Institue in Wurzburg. With sixty-three Chromo-Lithographic Plates, comprising 558 figures, and Numerous Engravings. Published by Wm. Wood & Co., New York. 1897. Price $3 net.

This is the sixth in a reform series issued by the publisher. The publishers say of them: "That for accuracy, beauty and compactness, is believed to be superior to anything heretofore published." In this statement we most heartily coincide. The work sets out with sixty-three full page chromo-lithographs, fully illustrating many of the most important bacteria in all stages of culture and development, with explanatory text on page facing the plate. Then follows about one hundred pages of the essentials of bacteriology closing with a timely chapter on technique.

The work will be of very great value to those who have a microscope and pursue their own investigations, as well as those who never having made a technical study of bacteriology desire a simple and systematic eliminary treatise with which to begin the study.

We have not seen the preceding volumes of the series, but if they are in every way equal to the volume before us they are worth many times their price. The only criticism that can be justly made is against the cloth binding. Such excellent specimens of art should be bound in durable leather.

JOHN KENDRICK BANGS' NEW STORIES.—John Kendrick Bangs' newest stories are to appear in *The Ladies Home Journal.* They are called "Stories of a Surburban Town." There are several, and each will relate some droll incidents in the life of a small town which every "surburbanite" will instantly appreciate and enjoy laughing over.

---

THE *Living Age*, in its issue for October 1st, is to begin a new serial story, translated for its pages from the French of Th. Bentzon, (Mme. Blanc). The story is entitled "Constance" and it is the study of the life of a young girl. Important ethical questions, especially that of divorce, are touched upon, and the story has a high moral purpose. The translation is made by Mrs. E. W. Latimer, and is authorized by Mme. Blanc.

---

THE *American Monthly Review of Reviews* for October gives special attention to the developments of the past month in international politics and to the lessons of the Spanish-America war. The editor, in the department of "The Progress of the World," discusses the attitude of the Spanish people toward peace conditions, the new relations between Germany and England, the Czar's proposition for disarmament, the Dreyfus case in France, England's reopening of the Soudan, and other serious problems confronting the European powers. Important contributed articles review President McKinley's course in the conduct of the war to a successful close and the deficiencies in our administrative machinery revealed by the fatal delays and break-downs in the medical and subsistence departments of army management.

---

THE University of Texas has just issued its annual catalogue. It contains 287 pages. An illustrated hand-book is sent out with it containing half-tone engravings of the buildings, grounds, etc. During the past year the University has enrolled 800 students, representing one foreign country, seven states and more than one hundred different counties of Texas. The graduating class in literature, law, medicine, pharmacy and nursing, numbered more than one hundred students. Six graduate students have won fellowships this year in Northern Universities. These fellowships pay from $300 to $600 annually without requiring any labor in return, and are open to competition of the world. More than 15C young ladies were in attendance.

The faculty consists of seventy-one officers and instructors and is unsurpassed in thorough scholarship, teaching power and breadth of learning.

A catalogue may be had by addressing John A. Lomax, Registrar, Austin, Texas.

---

**In an Early Day.**—Dr. Joseph Price, of Philadelphia, in his address as chairman of the Section on Gynecology at the Denver meeting of the American Medical Association (*Journal of the American Medical Association*) quotes the following from Dr. John Light Atlee, of 1843, which shows the spirit of abdominal surgery at that early day: "It is well known that from the earliest period of ovariotomy in Philadelphia down to the present time, it has been my invariable custom to invite members of the profession to witness the operation, in order that they might be able to form a proper opinion of its character and to judge of its propriety. There was not a prominent medical gentleman in this city that had not such an opportunity. It was a rare circumstance, during the probationary stage of the operation, for any one to accept the invitation cordially and gratefully. Some did so coldly, as if conferring a favor upon me, others politely declined, others positively refused and emphatically condemned the operation, while others regarded the invitation as an insult. Gentlemen who were bold enough to witness the operation were even directly accused by their professional acquaintances of being *particeps criminis* in committing murder, notwithstanding these murdered patients recovered. Some high in the profession, against all ethical consideration, would call upon the patients who had finally decided upon the operation, for the purpose of warning them against me and certain death. The colleges proclaimed fiercely against the operation as unjustifiable and criminal." Dr. Price quotes from Professor Meigs, who emphatically expressed himself, as follows: "I detest all abdominal surgery. I am free to say that I look upon all operations for the extirpation of the diseased ovaries as not to be justified by the most fortunate issue in any ratio whatever of the cases, or in other words, not to be justified by any amount of success."

